# Undernutrition *in utero* increases susceptibility to prostate neoplasias in adult rat after steroid exposure

**DOI:** 10.1186/1753-6561-7-S2-P32

**Published:** 2013-04-04

**Authors:** Jaqueline C Rinaldi, Sérgio L Felisbino

**Affiliations:** 1 Morphology Department, UNESP - Sao Paulo State University, Botucatu, SP, Brazil

## Background

Maternal protein restriction during pregnancy promotes several alterations in the progeny. Previous studies showed that androgen/estrogen imbalances during perinatal period, can program prostate cells to develop prostate lesions in adult life after a second insult. This study aimed to investigate prostate diseases susceptibility in adult rat offspring which underwent *in utero* low protein diet and were chronically exposed to low doses of estrogen and testosterone in adult life.

## Material and methods

16 weeks-old Wistar rats (n=48) that received *in utero* normal protein diet (NP group, 17% protein) or low protein diet (LP group, 6% protein) were subjected to 17-beta estradiol+testosterone administration (subcutaneous implant, NPH and LPH groups) for 16 weeks. The animals were killed at age of 35 week and the ventral (VP) and dorsolateral prostate (DLP) was excised, weighted and processed for histochemical, morphometrical and immunohistochemical (AR, Ki67, p63, beta-catenin, laminin and GSTP) analyses.

## Results

Both VP and DLP weight from NPH group were higher than LPH group. Serological data showed that estradiol levels were similar in both groups, but testosterone levels were lower in the LPH male offspring. Morphometric analysis verified a decrease in the height of prostatic epithelium, apoptotic index and an increase of proliferation index in LPH group compared to NPH group. The incidence of prostatitis and prostatic intraepithelial neoplasia was higher in VP and DLP of LPH group. However prostate cancer was not observed.

## Conclusion

Maternal protein restriction alters adult prostate response to androgen/estrogen handling and also interferes in adult prostate susceptibility to diseases.

## Financial support

FAPESP (2009/50204-6).

